# Cell-specific expression of tryptophan decarboxylase and 10-hydroxygeraniol oxidoreductase, key genes involved in camptothecin biosynthesis in *Camptotheca acuminata *Decne (Nyssaceae)

**DOI:** 10.1186/1471-2229-10-69

**Published:** 2010-04-19

**Authors:** Alessio Valletta, Livio Trainotti, Anna Rita Santamaria, Gabriella Pasqua

**Affiliations:** 1 Department of Plant Biology, "Sapienza" University of Rome, Piazzale Aldo Moro 5, 00185 Rome, Italy; 2 Department of Biology, University of Padua, Via Trieste 75, 35121 Padua, Italy

## Abstract

**Background:**

*Camptotheca acuminata *is a major natural source of the terpenoid indole alkaloid camptothecin (CPT). At present, little is known about the cellular distribution of the biosynthesis of CPT, which would be useful knowledge for developing new strategies and technologies for improving alkaloid production.

**Results:**

The pattern of CPT accumulation was compared with the expression pattern of some genes involved in CPT biosynthesis in *C. acuminata *[i.e., *Ca-TDC1 *and *Ca-TDC2 *(encoding for tryptophan decarboxylase) and *Ca-HGO *(encoding for 10-hydroxygeraniol oxidoreductase)]. Both CPT accumulation and gene expression were investigated in plants at different degrees of development and in plantlets subjected to drought-stress. In all organs, CPT accumulation was detected in epidermal idioblasts, in some glandular trichomes, and in groups of idioblast cells localized in parenchyma tissues. Drought-stress caused an increase in CPT accumulation and in the number of glandular trichomes containing CPT, whereas no increase in epidermal or parenchymatous idioblasts was observed. In the leaf, *Ca-TDC1 *expression was detected in some epidermal cells and in groups of mesophyll cells but not in glandular trichomes; in the stem, it was observed in parenchyma cells of the vascular tissue; in the root, no expression was detected. *Ca-TDC2 *expression was observed exclusively in leaves of plantlets subjected to drought-stress, in the same sites described for *Ca-TDC1*. In the leaf, *Ca-HGO *was detected in all chlorenchyma cells; in the stem, it was observed in the same sites described for *Ca-TDC1*; in the root, no expression was detected.

**Conclusions:**

The finding that the sites of CPT accumulation are not consistently the same as those in which the studied genes are expressed demonstrates an organ-to-organ and cell-to-cell translocation of CPT or its precursors.

## Background

*Camptotheca acuminata *Decaisne (Nyssaceae) is a deciduous tree native to south China and Tibet, where it is known as "*Xi Shu*" or "Happy Tree". *C. acuminata *is a main natural source of the terpenoid indole alkaloid (TIA) camptothecin (CPT), which was first isolated in 1966 by Wall and coworkers [1]. CPT has received great attention for its remarkable antitumor activities, which result from its ability to interact with DNA topoisomerase I [2,3]. In 1996, irinotecan [4] and topotecan [5], two semi-synthetic derivatives of CPT, were approved by the U.S. Food and Drug Administration (FDA) for treating colorectal and ovarian cancer. Other CPT derivatives, such as 9-nitroCPT and 9-aminoCPT, have also shown remarkable potential in the treatment of cancer.

**Figure 1:**
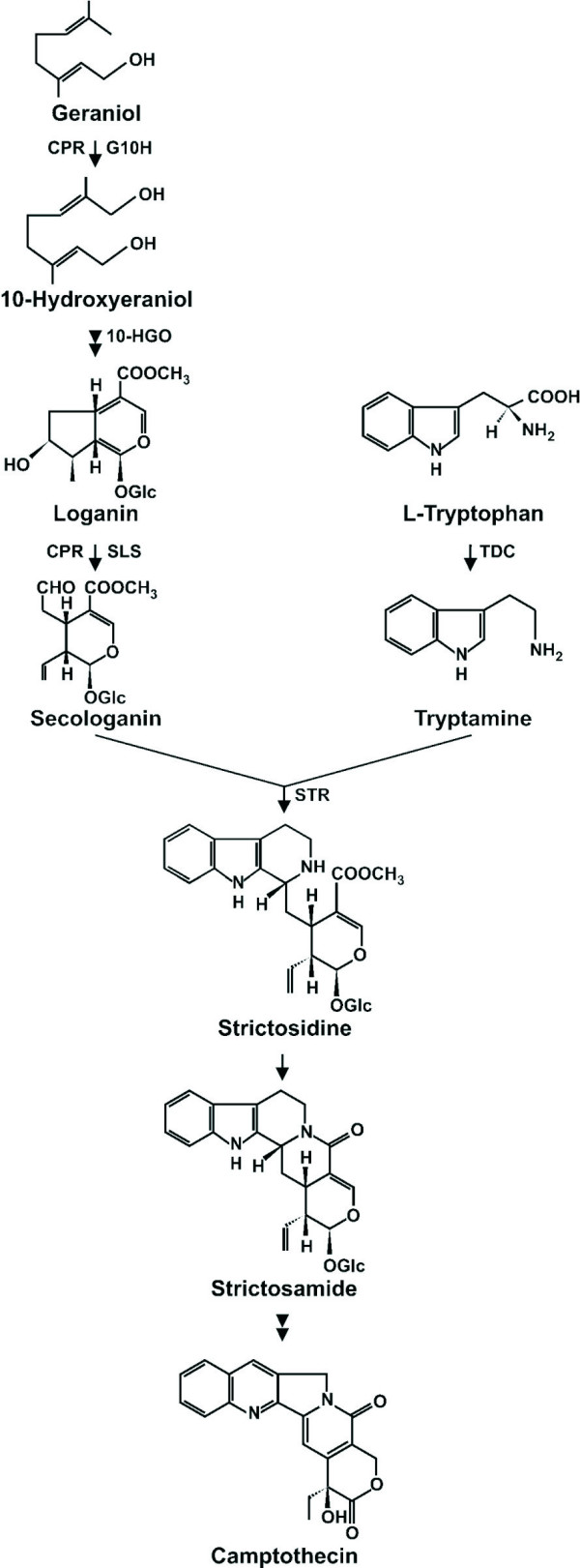
**Biosynthesis of camptothecin**. Tryptophan decarboxylase (TDC); geraniol 10-hydroxylase (G10H); NADPH:cytochrome P450 reductase (CPR); secologanine synthase (SLS); strictosidine synthase (STR). Double arrows indicate the involvement of multiple enzymatic steps.

TIAs are a broad group of alkaloids which include the anti-cancer compound vinblastine, the rat poison strychnine, and the anti-malarial drug quinine [6]. The precursors for TIA synthesis derive from the shikimate and mevalonate pathways, which supply the indole tryptamine and the iridoid secologanin, respectively (Figure 1). Tryptamine is synthesized from tryptophan (Trp), a step catalysed by tryptophan decarboxylase (TDC), whereas secologanin is derived from loganin, which is synthesized from the monoterpenoid 10-hydroxygeraniol, a step catalysed by 10-hydroxygeraniol oxidoreductase (10-HGO) [7]. The condensation of tryptamine and secologanin results in the formation of strictosidine, the common precursor for TIAs [8,9], which is then converted into strictosamide [10]. The steps following strictosamide formation have not been clearly defined, although some hypotheses have been formulated [10].

CPT accumulates in all organs of the *C. acuminata *plant, although the CPT content is higher in young leaves [11-13] and mature fruit [13]. At the cellular level, it accumulates in crystalline form in glandular trichomes, which are localised on both the leaf and young stem and in some specialized cells (segregator idioblasts), which are localised in parenchymatic and epidermal tissues [14]. The vacuole is the subcellular compartment in which CPT is stored [14], as generally occurs for alkaloids and many secondary metabolites [15].

However, little is known about the sites of CPT biosynthesis in the plant. In recent years, some genes involved in the very early steps of the biosynthetic pathway have been investigated. Lu et al. [16] cloned and characterized the α-subunit of anthranilate synthase from *C. acuminata *(*Ca-ASA*), which catalyzes the first reaction of the indole pathway. The expression pattern of *Ca-ASA *has been studied in transformed tobacco plants carrying the promoter of this gene fused with a GUS reporter gene. Lu and Mcknight [17] cloned and characterized the β-subunit of tryptophan synthase from *C. acuminata *(*Ca-TSB*); *Ca-*TSB mRNA and protein were detected in all organs of the plant, and their abundance was correlated with CPT accumulation. Through tissue printing technique, it has been demonstrated that in all shoot organs *Ca-TSB *is mainly expressed in vascular tissues, whereas in the root it is mainly expressed in the subepidermal cortex.

López-Meyer and Nessler [18] isolated and characterised two autonomously regulated genes encoding TDC (*Ca-TDC1 *and *Ca-TDC2*) in *C. acuminata*. TDCs are key enzymes in the biosynthetic pathway of TIAs because they link primary to secondary metabolism by converting Trp into tryptamine. Tryptamine is a precursor for the biosynthesis of both indole acetic acid (IAA) [19] and TIAs [20]. The relationship between TDC and TIA biosynthesis has been extensively studied in *Catharanthus roseus*. In cell cultures of this species, treated with biotic and abiotic elicitors [21] or transferred to an alkaloid production medium [22], the activity of TDC has been shown to be correlated with the accumulation of TIAs. In *C. roseus *roots cultured *in vitro*, TDC activity was correlated with vindoline accumulation [23]. TDC is also highly expressed in developing plantlets of *C. roseus*, and the exogenous application of signalling molecule methyl jasmonate enhances both TDC activity and TIA accumulation [24]. López-Meyer and Nessler [18] observed that *Ca-TDC1 *is expressed at different levels in all organs of the plant, with the highest level in the shoot apex, which, besides being a main site of IAA synthesis, is also the main site of CPT accumulation [11]. In developing plantlets, the higher expression of *Ca-TDC1 *was observed at day 10 post-imbibition, 2 days before the peak of CPT accumulation; these data suggest that *Ca-TDC1 *"may be part of a developmentally regulated chemical defence system". The expression of *Ca-TDC2 *was detected exclusively in leaf disks elicited with yeast extract and methyl jasmonate; thus this gene seems to be a "part of a defence system induced during pathogen challenge" [18].

Frequently, the synthesis of alkaloids involved in chemical plant defence against pathogen attack is also stimulated by abiotic stress (e.g., drought and mechanical and nutritional stress) [25]. It has been reported [26,27,14] that *C. acuminata *responds to different types of environmental stress with an increase in CPT biosynthesis.

The objective of the present study was to determine whether CPT accumulation and biosynthesis occur in the same cellular sites in *C. acuminata*. To this end, the accumulation pattern of CPT was compared with the expression pattern of *Ca-TDC1*, *Ca-TDC2*, and *Ca-HGO *genes. CPT accumulation was detected by HPLC and fluorescence microscopy, whereas gene expression was investigated by *in situ *hybridization. Both the accumulation of CPT and the expression of *Ca-TDC *and *Ca-HGO *genes at the cellular level were investigated in samples collected from plants at different stages of development and subjected to drought-stress.

## Results

### CPT accumulation in the shoot apex and young leaves

**Figure 2:**
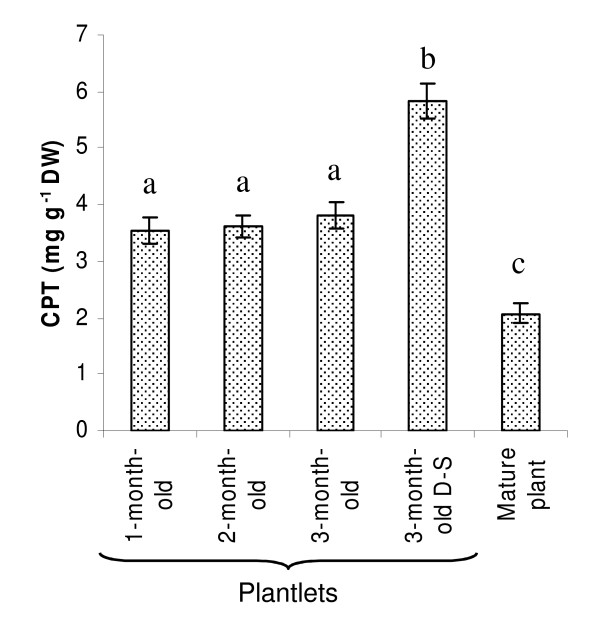
**CPT concentration in the shoot apex and in the first 4 leaves of plantlets and mature plants of *Camptotheca acuminata*, and the effect of drought-stress (D-S) on CPT production**. Each value represents the means of three independent determinations; the vertical lines and different letters above the bars indicate standard errors (SE) and statistically significant differences (*P *≤ 0.05) between concentrations.

CPT content in the shoot apex and in the first four leaves of mature plants and plantlets was evaluated by means of HPLC analysis (Figure 2). CPT concentration in the plantlets (subjected and not to drought-stress) (3.54-3.81 mg g^-1 ^D.W.) was higher than in the mature plants (2.08 mg g^-1 ^D.W.). No significant differences were observed when comparing one-, two-, and three-month-old plantlets. In the three-month-oldplantlets subjected to drought-stress, CPT accumulation (5.84 mg g^-1 ^D.W.) was significantly greater than in the unstressed three-month-old plantlets.

### Cell- and tissue-specific accumulation of CPT

**Figure 3:**
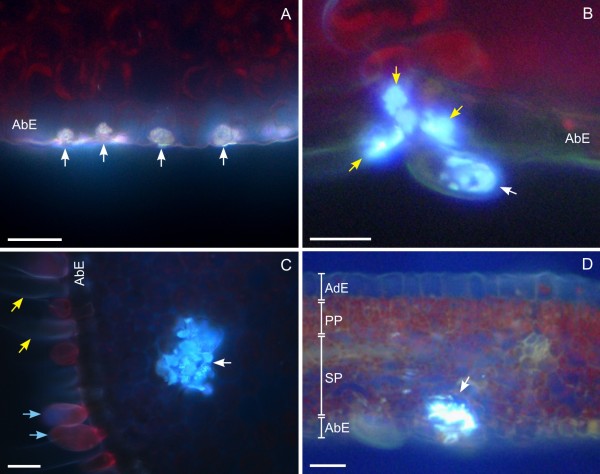
**Optical micrographs of fresh leaf cross sections of *Camptotheca acuminata *3-month-old plantlets observed under UV-light**. (A) Epidermal cells accumulating CPT (arrows) localized on the abaxial side of the leaf midrib; (B) accumulation of CPT in a glandular trichome (white arrow) and in some epidermal cells (yellow arrows) surrounding it; (C) group of segregator idioblasts (white arrow) containing CPT crystals localized in the leaf mesophyll, at midrib level; in the section, glandular trichomes (light-blue arrows) and non-glandular trichomes (yellow arrows) can be observed; (D) CPT accumulation in a group of segregator idioblasts localized in the spongy parenchyma adjacent to the abaxial epidermis. Abaxial Epidermis (AbE); Adaxial Epidermis (AdE); Palisade Parenchyma (PP); Spongy Parenchyma (SP). (A) bar = 5 μm; (B, C) bar = 10 μm; (D) bar = 50 μm.

**Figure 4:**
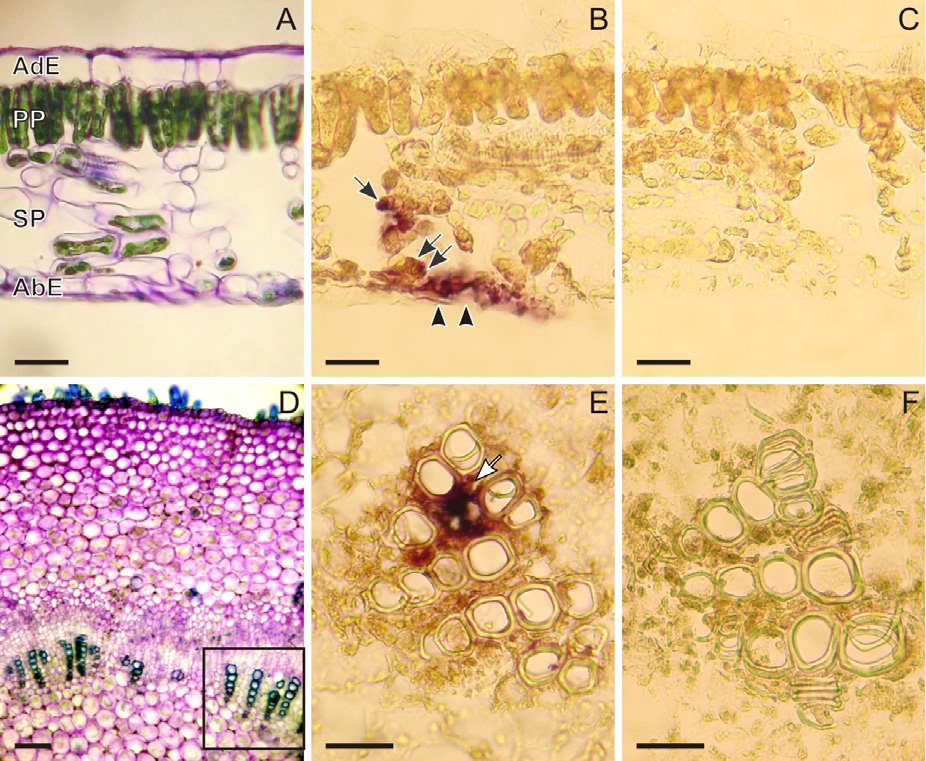
***Ca-TDC1 *expression in the leaf and stem of 3-month-old *Camptotheca acuminata *plantlets**. Fresh cross sections of the leaf (A) and primary stem (D) stained with 0.1% toluidine blue to show the anatomical structure. Paraffin-embedded cross sections of leaves (B and C) and primary body of the stem (E and F), treated with *Ca-TDC 1 *antisense (B and E) and sense (C and F) digoxigenin-labelled probes. The square in the bottom right corner of D indicates a vascular bundle, that is, the part of the stem section shown in E and F. The hybridization signals in the leaf treated with antisense probe (B) are present in some spongy parenchyma cells (arrow), in parenchymatic subepidermal cells (double arrows), and in two epidermal cells (arrowheads). In the stem treated with antisense probe (E), hybridization signals are present in parenchyma cells associated with vascular bundles (arrow). No hybridization signals are present in the sections of leaf (C) and stem (F) treated with sense probe. Abaxial Epidermis (AbE); Adaxial Epidermis (AdE); Palisade Parenchyma (PP); Spongy Parenchyma (SP). (A, B, C, E, F) bar = 50 μm; (D) bar = 100 μm.

CPT accumulation was visualized under a light fluorescent microscope on fresh sections of the first four leaves of plantlets and on mature leaves. *C. acuminata *has simple, dorsoventral, elliptical leaves. The leaf epidermis, on both the adaxial and abaxial side, is composed of a single layer of thin-walled cells, whereas the mesophyll is composed of a single layer of elongated palisade parenchyma on the adaxial side and a multiple layer of spongy parenchyma on the abaxial side (Figures 3D and 4A). In the leaf, as in the young stem, both glandular trichomes (GT) and non-glandular trichomes are present, and their density decreases with the age of the organ [13,14,28]. In the leaf, as in the young stem, unbranched, non-articulated laticifers are associated with the veins [29].

In all of the samples, light-blue autofluorescent crystals of CPT were present in some epidermal idioblasts (EI) (Figure 3A), in some GTs (Figure 3B), and in groups of idioblast cells (GIC) (Figure 3C, D), each of which consisted of 2-10 cells localized in parenchymatic tissues and not organized to form a multi-cellular secretory structure. CPT accumulation was not observed in the laticifers in either the leaf or the stem.

The number of EIs with CPT decreased with increasing age of the plant, from an average of 32.81 EIs in a 5-mm section of a one-month-old plantlet to an average of 7.51 EIs in a 5-mm section of the mature plant. No significant differences were observed when comparing stressed and unstressed plantlets (Table 1).

**Table 1:** Cellular sites of CPT accumulation in cross sections (about 5 mm in length) through the midrib of a leaf

		1-month-old plantlet	2-month-old plantlet	3-month-old plantlet	3-month-old plantlet (drought-stress)	Mature plant
**EIs**	Tot	230 ± 9.01	223 ± 11.24	190 ± 7.03	190 ± 8.16	103 ± 4.26
	N	32.81 ± 0.22 (a)	29.20 ± 0.61 (b)	19.93 ± 0.36 (c)	21.10 ± 0.23 (c)	7.51 ± 0.39 (e)
**GTs**	Tot	12.31 ± 1.12	8.96 ± 1.21	6.98 ± 1.95	9.01 ± 1.43	4.12 ± 2.33
	N	3.22 ± 1.14 (a)	1.87 ± 1.05 (b)	1.26 ± 1.83 (c)	2.01 ± 1.25 (d)	0.54 ± 1.98 (e)
**GICs**	N	3.02 ± 1.21 (a)	2.23 ± 1.10 (b)	1.43 ± 0.82 (c)	1.17 ± 0.67 (c)	0.42 ± 0.32 (e)

(N) average number of cells or trichomes accumulating CPT per section; (Tot) average number of cells or trichomes per section. Different letters indicate statistically significant differences (*P *≤ 0.05) between values. (EC) epidermal cells; (GT) glandular trichomes; (IC) idioblast cells accumulating CPT.

Although the GTs are present on both sides of the leaf, those containing CPT crystals were mostly localized on the abaxial side. In some cases, CPT accumulation was also observed in epidermal cells surrounding the GTs (Figure 3B). In unstressed plantlets, the average number of GTs with CPT crystals decreased with age, from 3.22 per 5-mm section for three-month-old plantlets to 1.26 for one-month-old plantlets (Table 1). In three-month-old plantlets subjected to drought-stress, the number of GTs with CPT (average of 2.01 per section) was significantly higher, compared to same-age unstressed plantlets (1.26 per section) (Table 1). In the mature plant, the average number of GTs with CPT accumulation (0.54 per section) was significantly lower than that in the plantlets; the total number of leaf GTs (with and without CPT accumulation) was also much lower in mature plants than in plantlets.

Most GICs were present in the parenchyma tissue surrounding the midrib (Figure 3C), although they were also observed in the mesophyll of the leaf lamina, in both the palisade and spongy parenchyma (Figure 3D). The number of GICs decreased with the age of the plant, from an average of 3.02 per section in one-month-old plantlets to 0.42 per section in the leaf of the mature plant; no statistically significant differences were observed between stressed and unstressed plantlets (Tab. 1).

In the stem and root, CPT accumulation was observed in the same cellular sites previously described by Pasqua et al. [14], and no differences were found when comparing stressed and unstressed plants.

### Cell- and tissue-specific distribution of *Ca-TDC1 *transcripts

In the leaf, *Ca-TDC1 *gene expression at the cellular level did not completely correspond with the pattern of CPT accumulation described above. Hybridization signals were only observed in some EIs and in some GICs localized in both the spongy (Figure 4B) and palisade parenchyma. These groups of cells were sometimes in contact with the adaxial or abaxial epidermis (Figure 4B). The cells in which *Ca-TDC1 *expression was detected did not differ in terms of shape or size from the cells of surrounding tissues. Surprisingly, no *Ca-TDC1 *expression was detected in the GTs on either the abaxial or adaxial side.

In the stem, in both the primary and secondary body, *Ca-TDC1 *expression was observed in the vascular tissues, specifically, in the parenchymatic cells surrounding the xylem cells (Figure 4E). No hybridization signals were observed in the GTs, EIs, the cortex or the pith. No differences were observed between plantlets and mature plants or between stressed and unstressed plantlets.

In the primary and secondary body of the root, no hybridization signals for *Ca-TDC1 *expression were detected.

**Figure 5:**
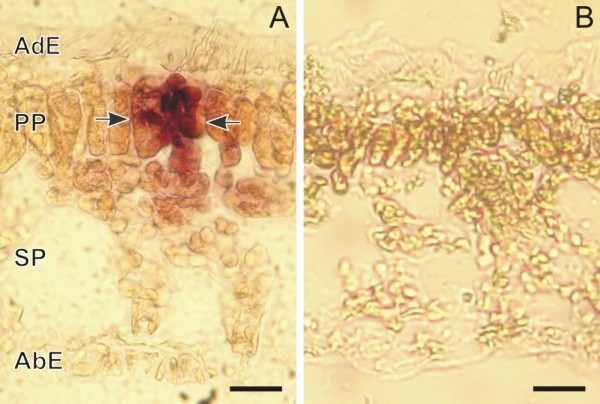
***Ca-TDC2 *expression in the leaf *Camptotheca acuminata *plantlets subjected to drought-stress**. Paraffin-embedded cross sections of leaves treated with *Ca-TDC 2 *antisense (A) and sense (B) digoxigenin-labelled probes. The hybridization signals are present in some palisade parenchyma cells (arrows). Abaxial Epidermis (AbE); Adaxial Epidermis (AdE); Palisade Parenchyma (PP); Spongy Parenchyma (SP). (A, B) bar = 50 μm; (D) bar = 100 μm.

The expression of the *Ca-TDC2 *gene was only observed in the leaves of plantlets subjected to drought-stress. In these plantlets *Ca-TDC1 *transcripts were also detected with the same cellular localization observed in unstressed plantlets. As found for *Ca-TDC1*, *Ca-TDC2 *expression was observed in GICs, localized in the spongy and palisade parenchyma (Figure 5). No hybridization signals were observed in the EIs, the GTs, or in the tissues of the vascular bundles.

### Cell- and tissue-specific distribution of *Ca-HGO *transcripts

**Figure 6:**
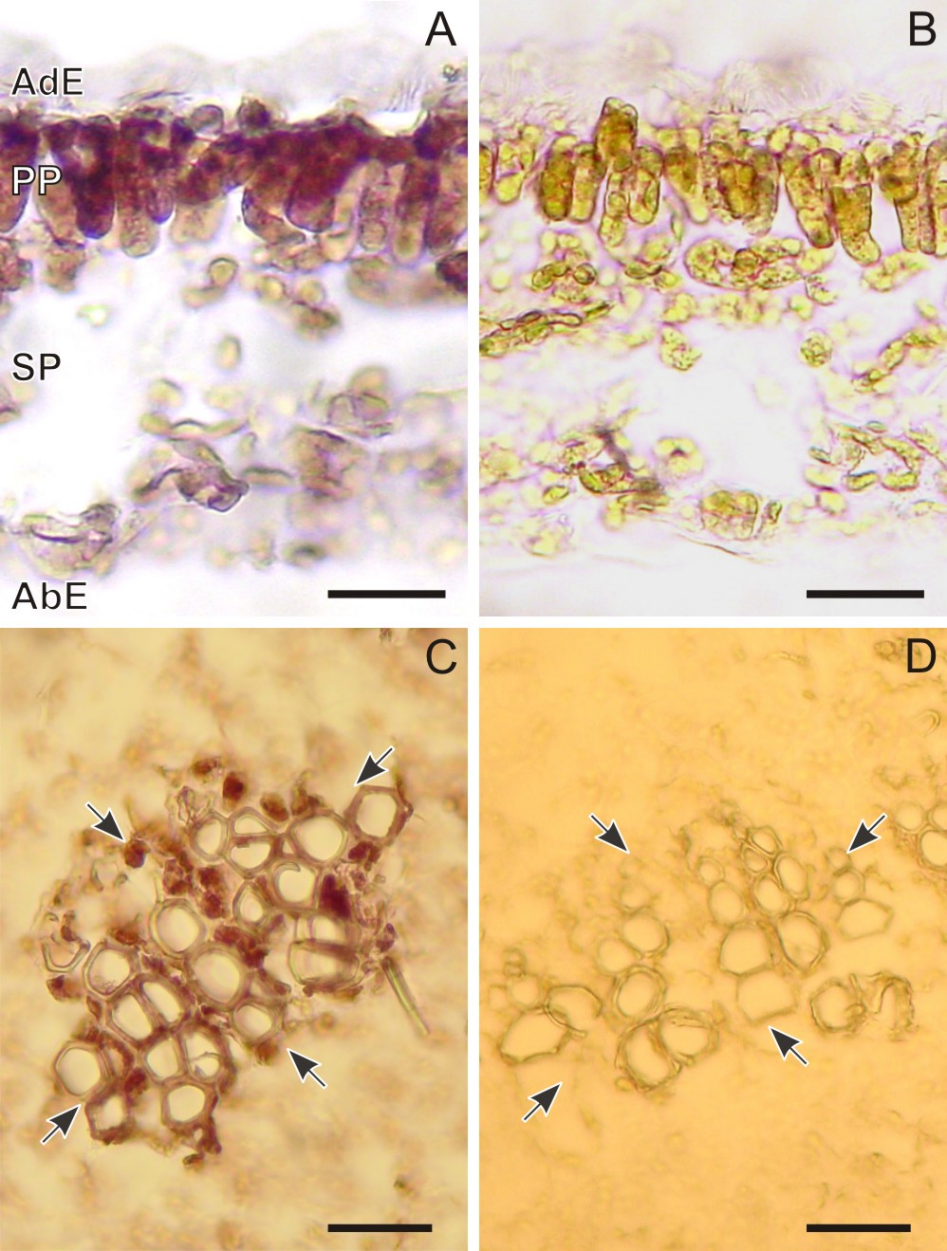
***Ca-HGO *expression in the leaf and stem of 3-month-old *Camptotheca acuminata *plantlets**. Paraffin-embedded cross sections of leaves (A and B) and primary body of the stem (C and D), treated with *Ca-HGO *antisense (A and C) and sense (B and D) digoxigenin-labelled probes. The hybridization signals in the leaf treated with antisense probe (A) are present in all mesophyll cells. In the stem treated with antisense probe (C), hybridization signals are present in parenchyma cells associated with vascular bundles (black arrows). No hybridization signals are present in the sections of leaf (B) and stem (D) treated with sense probe. Abaxial Epidermis (AbE); Adaxial Epidermis (AdE); Palisade Parenchyma (PP); Spongy Parenchyma (SP). Bar = 50 μm.

In leaves, *Ca-HGO *expression was observed in the chlorenchyma cells; differently from *Ca-TDC1 *and *Ca-TDC2*, whose expression was observed in groups of cells, *Ca-HGO *expression was distributed throughout the entire mesophyll (Figure 6A). No hybridization signals were detected in the EIs or the GTs.

In the stem, *Ca-HGO *expression, like *Ca-TDC1 *expression, was observed in the parenchyma cells localized in the vascular bundles (Figure 6C). No hybridization signals were observed in the EIs, the GTs, the cortex, or the pith. No differences were observed between the stem of plantlets and the mature plant or when comparing the stems of stressed and unstressed plants.

In the roots of the plantlets and the mature plant (both the primary and secondary structure), no hybridization signals were observed.

**Figure 7:**
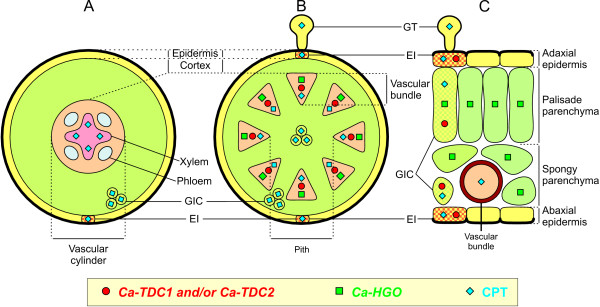
**Diagram displaying the expression of *Ca-TDC1*, *Ca-TDC2*, and *Ca-HGO *genes, and CPT accumulation in different cells, tissues, and organs**. Root (A), stem (B), and leaf (C). Epidermal Idioblast (EI); Glandular Trichomes (GT); Group of Idioblast Cells (GIC).

In Figure 7 the sites of TDC/HGO expression and CPT accumulation in the different organs and tissues are summarizes.

## Discussion

In the present study, the accumulation pattern of CPT in *C. acuminata *was compared with the expression pattern of *Ca-TDC1*, *Ca-TDC2*, and *Ca-HGO *genes, which are involved in TIA biosynthesis. Both the accumulation of CPT and the expression of *Ca-TDC *and *Ca-HGO *genes at the cellular level were investigated in samples collected from plants at different stages of development and subjected to drought-stress, since it is well known that the biosynthesis, transport, and accumulation of plant alkaloids are strongly associated with development and with biotic and abiotic environmental stimuli [6,24,30,31].

The first step of this experiment was to determine whether drought-stress increases CPT production. In a study on the relationship between drought-stress and CPT production in *C. acuminata *[27], only plants whose seeds came from certain geographic locations showed increased CPT production in response to drought-stress. In our plants, chemical analyses confirmed that drought-stress induced a significant increase in CPT production. Other studies have shown that CPT production in *C. acuminata *is also enhanced by other types of adverse growing conditions, such as heavy shade [32], heat shock [33], pruning [14], and nutritional stress [14,34]. These results support the hypothesis that CPT plays a role in the chemical defence of the plant. Pathogenic and herbivorous attacks can result in the loss of cells, tissues, or entire organs, which are replaced with more difficulty in plants with retarded growth; for this reason, these plants require greater defences than the same species grown under favourable environmental conditions. Although the hypothesis that CPT is involved in chemical defence has not been directly proven [35], it is supported by indirect evidence, such as the lack of damage caused by insects and pathogens in *C. acuminata *plantations in the USA [36]. It is also supported by our finding that the number of accumulation sites decreased with plant age, as did the CPT content, which is consistent with the results of other studies [17,18,37]. Moreover, the role of other alkaloids in chemical defence has been proven for other plant species [6,38-41].

The second step of this experiment was to determine whether the quantity of CPT was associated with the accumulation pattern at the cellular level. In all of the samples, fluorescent microscope analyses showed that CPT accumulation occurred in the same cellular sites, in particular, in the GTs (in the leaf and young stem), in some EIs (in the leaf, stem, and root), and in the GICs (in the parenchymatic tissues of the leaf, stem, and root). CPT accumulation was not observed in all of the GTs, which could be explained in two ways: i) only some of the GTs are able to produce and/or accumulate CPT; or ii) all of the GTs are able to produce and/or accumulate CPT, but some of them do it constitutionally, whereas others do so exclusively when induced by specific stimuli. The latter hypothesis is supported by the finding that the percentage of CPT accumulating GTs was much higher in the plantlets subjected to drought-stress, compared to same-age unstressed plantlets.

To identify the sites of the early stages of CPT biosynthesis at the cellular level and determine whether these sites are the same as those of CPT accumulation, the cell-specific localization of *Ca-TDC *and *Ca-HGO *expression was investigated. In several species, alkaloid biosynthesis occurs in cells, tissues and organs that are different from those where accumulation takes place. For example, in Solanaceae species, the tropane alkaloids are first synthesised in the root and then transported, through the vascular tissue, to the bud and leaf, which are the main sites of accumulation [24,30,42]. One way of investigating the compartmentalisation of alkaloid biosynthesis is to localize the expression of genes involved in their biosynthetic pathway. In *C. roseus*, RNA *in situ *hybridization combined with immunocytolocalization techniques has demonstrated that the genes involved in the early stages of vindoline biosynthesis (*TDC *and *STR1*) are expressed in the epidermis of the stem, leaf, and flower bud, and in the apical meristem of the root tip, whereas the genes involved in the terminal stages (*D4H *and *DAT*) are expressed in the laticifer and idioblast cells of the leaf, stem and flower bud [24]. These results demonstrate that vindoline biosynthesis involves the participation of different cell types and that it requires the intercellular translocation of the pathway intermediates.

Several studies carried out on *C. acuminata *[18] and *C. roseus *[43,22] have shown that an increase in TIA biosynthesis is accompanied by an increase in TDC activity; thus these enzymes seem to play a leading role in the regulatory control of the TIA biosynthetic pathway. In our study, the hybridization signals obtained with *Ca-TDC1 *and *Ca-TDC2 *probes were very intense and circumscribed to single cells or small groups of cells; in the surrounding tissues, no hybridization signals were observed, not even weak signals. Since TDC enzymes are involved in the biosynthesis of not only TIAs but also other metabolites (e.g., proteins and, in some species, IAA), it was surprising that in our study *Ca-TDC *expression was limited to specific cells. It is possible that these genes are expressed in the majority of cells but that the expression levels are too low to be detected by *in situ *hybridization, possibly because of the strong dilution factor of the probes used.

In all of the samples, *Ca-TDC1 *transcripts were detected in the leaf and stem. In these organs, some of the cellular sites with *Ca-TDC1 *showed a similar localization with respect to CPT accumulation, that is, the epidermal and parenchymatic tissues. No *Ca-TDC1 *transcripts were observed in the GTs, but interestingly, hybridization signals were sometimes detected in the EIs surrounding them, which are the same cellular sites in which CPT accumulation was sometimes observed. These data suggest that CPT might be biosynthesised in these EIs and then transported to the GTs, which serve as sinks for CPT, even if they are not capable of biosynthesising this alkaloid.

In none of the analysed samples was *Ca-TDC1 *expression detected in the root, although CPT does accumulate in this organ. Previous results demonstrated that no CPT was produced by roots regenerated *in vitro *from leaf explants; by contrast, roots originating from micro-cuttings (with axillary buds) accumulated CPT, though at a low concentration [44]. López-Meyer and Nessler [18] detected *Ca-TDC1 *expression in all parts of one-year-old *C. acuminata *plants, including the root, although in this organ the expression level was very low. It is possible that this gene was also expressed in our plants but that the amount of the transcripts was too low and delocalised to be detected by *in situ *RNA hybridization. Lu et al. [16] and Lu and McKnight [17] cloned and characterized, respectively, the α-subunit of anthranilate synthase (ASA) and the β-subunit of tryptophan synthase (TSB) from *C. acuminata*, enzymes involved in the indole pathway. They demonstrated that both ASA and TSB enzymes were expressed in the root of *C. acuminata *at very low levels compared to the other parts of the plant. Although the root is a site of CPT accumulation, the above-mentioned results suggest that this organ is not a site of CPT biosynthesis, at least for the early stages of the biosynthetic pathway. This is in contrast with the opinion of other authors [11,28] who have hypothesized that this alkaloid may be completely synthesized in the root and then transferred to the shoot organs, such as occurs for tropane alkaloids and nicotine [24,30,42]. In another CPT-producing plant, *Ophyorrhiza pumila*, the highest *TDC *expression was detected in the root, which is the main site of CPT accumulation, and no expression was detected in the leaf, in which CPT accumulation is very low [7].

The *Ca-TDC2 *transcripts were observed exclusively in the leaf of plantlets subjected to drought-stress, and these samples *Ca-TDC1 *transcripts were also detected. López-Meyer and Nessler [18] did not observe *Ca-TDC2 *expression in unstressed plantlets at any point in their development; they induced the expression of this gene by eliciting *C. acuminata *leaf disks with yeast extract and methyl jasmonate, which did not affect *Ca-TDC1 *expression. Based on these results, the authors hypothesized that *Ca-TDC2 *is a part of an inducible defence system, whereas *Ca-TDC1 *is part of a developmentally regulated defence system.

The expression of *Ca-TDC2 *was detected both in the leaf and stem, in some EIs and ICs, as found for *Ca-TDC1*, yet the number of these cellular sites per section was higher than those in the sections treated with the *Ca-TDC1 *probe. In stressed plants, in addition to an increase in CPT, there was an increase in the number of cells with CPT accumulation. This suggests that *C. acuminata *possesses, in both the leaf and stem, specialised cells whose capacity to biosynthesize and accumulate CPT is activated exclusively in response to stress.

*Ca-HGO *gene was expressed in the leaf and stem but not in the root. In the stem, *Ca-HGO *transcript was observed in the same sites as *Ca-TDC 1 *and *2 *expression. In the leaf, *Ca-HGO *expression was detected in chlorenchyma cells, yet differently from that which was found for *Ca-TDC 1 *and *2*, it extended to the entire mesophyll and was not restricted to specific groups of cells.

The different localization of *Ca-HGO *and *Ca-TDC *transcripts reflects a different localization of iridoid and indole biosynthetic pathways, from which derived CPT intermediates (secologanin and tryptamine). The compartmentation of biosynthetic pathways implies that there is a cell-to-cell transport of these intermediates, and that they accumulate in cells where the late stages of CPT biosynthesis occur. Multi-cellular compartmentation has been demonstrated for other alkaloid-producing species [45], such as *Atropa belladonna*, *Hyoscyamus niger*, *Papaver somniferum*, *Thalictrum flavum*, and *Chatharanthus roseus*. In *C. roseus*, which has been the most widely studied species in terms of indole alkaloid biosynthesis, the early iridoid pathway occurs in adaxial phloem parenchyma cells of aerial organs, whereas the late stage of both the iridoid pathway and indole pathway occurs in epidermal cells [45].

## Conclusions

The obtained results demonstrate that the root is not involved in CPT biosynthesis, although it is a site of CPT accumulation. CPT biosynthesis requires the participation of different cell types localized in the leaf and stem, and the intercellular translocation of CPT or its precursors has been hypothesized. The cloning of the genes responsible for the last steps in CPT biosynthesis and the localization of their expression at the tissue and cellular level will help to solve the puzzle of the synthesis of this useful alkaloid.

## Methods

### Plant material and drought-stress

Plant samples were collected from *C. acuminata *plantlets (one, two, and three months old) and mature plants (about five years old) grown in pots with commercial soil in the greenhouse of the Botanical Garden of the University "Sapienza" of Rome (Italy). Some three-month-old plantlets were subjected to three cycles of drought-stress using the dry-down and recharge technique, as described by Liu and Dickmann [46].

### CPT extraction

The shoot apex and the first four leaves of the mature plants and plantlets were frozen with liquid nitrogen and powdered with a mortar and pestle. The powdered plant material (about 100 mg per sample) was extracted with methanol by sonication for 30 min at room temperature. The methanolic extract (50 ml) was then filtered and evaporated at 40°C in a vacuum using a rotavapor; it was then redissolved in HPLC-grade methanol (1 ml).

### HPLC analysis

The HPLC system (Waters, Milford, MA, USA) consisted of an HPLC pump (1525 Binary HPLC Pump), a reversed phase column (Symmetry C_18 _4.6 × 250 mm) and a detector (2487 Dual λ Absorbance Detector) for detecting CPT at 254 and 370 nm. The flow rate was 1 ml min^-1^, and the isocratic mobile phase consisted of water:acetonitrile (70/30, v:v). The identification and quantification of CPT was performed based on the retention time and absorbance spectra of CPT reference solutions (0.1, 0.05, 0.01, 0.05, 0.001 mg ml^-1^) (Sigma, St. Louis, MO, USA).

### Fluorescence microscopy

The cellular sites of CPT accumulation were detected by means of fluorescence microscopy, as previously reported by Pasqua et al. [14]. The histochemical analyses were carried out on the first four leaves, stem, and root of mature plants and on all plantlets. Samples were collected from plants, immediately embedded in agar (4%), and then sectioned (30-40 μm thickness) with a vibratome (TPI series 1000, St. Louis, MO, USA). Fresh sections were examined with a light microscope (Axioscop 2 Plus, Carl Zeiss Inc., Thornwood, NY, USA) equipped with a Zeiss UV-filter (BP 365 nm, LP 397 nm). CPT was recognised by examining the characteristic crystal morphology and the light-blue autofluorescence that this compound emits under UV-light [14,47]. For each sample, about 100 sections were analysed, and for each section, the number of cellular sites of CPT accumulation was counted.

### Probe synthesis

For the synthesis of the antisense and sense *Ca-TDC1 *(Acc. no.: U73656), *Ca-TDC2 *(Acc. no.: U73657) and *Ca-HGO *(Acc. no.: AY342355) RNA probes, gene-specific regions were amplified with the following primers: *Ca-TDC1-*for (5'-GCGGATGTTCTCCTGAAAGAG-3') and *Ca-TDC1*-rev (5'-GATAGGATGCGCAGCACAAC-3') for *Ca-TDC1*; *Ca-TDC2*-for (5'-CTAAACAACCGGCCCACACC-3') and *Ca-TDC2*-rev (5'-CATTTGGAGGCAATATTGGAG-3') for *Ca-TDC2*; and *Ca-HGO*-for (5'-ATGGGAGGGATGAAGGAGACACA-3') and *Ca-HGO*-rev (5'-ACCAAAGTTCGGAGGGCACAG-3') for *Ca-HGO*.

As regard the TDC genes, the amplified fragments correspond to the last 27 bp of the coding sequence and 219 bp of 3' UTR for a total of 245 bp and to 152 bp of the 5' UTR and the codon of the starting Met, for a total of 155 bp for *Ca-TDC1 *and *Ca-TDC2*, respectively. The selected fragments have a similarity index of only 34.2% (calculated with Wilbur-Lipman algorithm with the following standard parameters: ktuple: 3; Gap penalty: 3; Window: 30), thus too low to for each probe to cross-react with mRNAs transcribed from the other gene. Moreover, sequencing of PCR amplification products never yielded contaminations of a gene product in any reaction specific for the other.

All inserts were cloned in the pGEM-T easy vector (Promega, Heidelberg, Germany) and were sequenced to verify their identities. The gene-specific DNAs, used to synthesize the RNA probes, were prepared by PCR in a standard reaction, using, as templates, 100 pg of plasmid DNA, oligonucleotides pUC/M13 forward and reverse as primers, and 1 unit of Taq DNA polymerase. Sense and antisense digoxigenin-labelled probes were synthesized using, as templates, the PCR products, which contained the T3 (for sense RNA synthesis) and T7 (for antisense RNA synthesis) promoters, and following the manufacturer's instructions (Roche Molecular Biochemicals, Penzberg, Germany).

### Tissue fixation and embedding

Tissues were fixed in a solution of formaldehyde/acetic acid/ethanol (3:5:60, v/v/v) at 4°C overnight. The fixed material was dehydrated through an ethanol and *ter*-butanol series and then embedded in paraffin.

### *In situ *hybridization

The *in situ *hybridization was performed as described by Cañas et al. [48], with minor modifications. The paraffin-embedded samples were sectioned (8-10 μm) using a microtome (Zeiss). Sections were spread on Superfrost Plus slides (Fisher Scientific) treated with 2% (v/v) bind-sylane (Amersham) in acetone, dried for 24 h at 40°C and stored at - 20°C until use. To remove paraffin, the samples were subjected to two incubations of 20 min each in xylene; to rehydrate the sections, an ethanol series up to water was used. The sections were then briefly rinsed in 0.05 M Tris/HCl, pH 7.6, and incubated with 0.5 ml of proteinase K (1 μg ml^-1^) in 0.05 M Tris-HCl, pH 7.6, for 25 minutes at 37°C. The proteinase K was removed with two rinses at 4°C in DEPC-treated H_2_O. The sections were then treated with acetic anhydride in 85 mM TEA buffer, pH 8.0, and rinsed three times with water. The sections were then dehydrated using an alcohol series and dried. For hybridization, the sections were incubated at 45°C overnight with hybridization buffer, under the cover glasses. The hybridization buffer consisted of 100 ng ml^-1 ^digoxigenin-labelled RNA, 50% formamide, 300 mM NaCl, 10 mM Tris/HCl pH 7.5, 1 mM EDTA, 1× Denhards solution, 10% dextrane sulfate, 10 mM DTT, 200 ng ml^-1 ^tRNA and 100 μg ml^-1 ^poly (A). After hybridization, cover glasses were washed in 2× SSC at room temperature, and the sections were rinsed three times for 25 minutes in 0.2× SSC preheated at 50°C. Treatment with RNase A (20 μg ml^-1 ^in 500 mM NaCl/TE pH 8.0) was then performed at 30°C for 30 min. The sections were then stained overnight at room temperature with alkaline phosphatase-conjugated antidigoxigenin antibodies, according to the protocol of Boehringer, using NBT and X-phosphate as substrates.

For each sample, about 100 sections were obtained (50 treated with sense probes and 50 with antisense probes). For each section, the number of hybridization signals was counted.

### Statistical analysis

In all experiments, the significance of the differences between the mean values was tested using ANOVA and the Student-Neuman-Keuls test by SPSS software. Differences with *P *< 0.05 were considered as statistically significant.

## Abbreviations

**HGO**: 10-hydroxygeraniol oxidoreductase; **CPR**: NADPH: cytochrome P450 reductase; **CPT**: camptothecin; **D-S**: drought stress; **DW**: dry weight; **EC**: epidermal cell; **FDA**: Food and Drug Administration of the USA; **GIC**: parenchymatic idioblast cell; **GT**: glandular trichome; **HPLC**: high performance liquid chromatography; **SE**: standard error; **SLS**: secologanine synthase; **STR**: strictosidine synthase; **TDC**: tryptophan decarboxylase; **TIA**: terpenoid indole alkaloid; **Trp**: tryptophan; **TSB**: β-subunit of tryptophan synthase.

## Authors' contributions

AV cloned Ca-TDC1, Ca-TDC2 and Ca-HGO genes and carried out chemical analyses and histological analyses. LT synthesised the riboprobes for *in situ *hybridization experiments and, together with AV and GP, he drafted/constructed the manuscript. AS carried out, together with AV, the *in situ *hybridization experiments. GP supervised all research.
